# First WGS Characterization of *Streptococcus suis* Isolated From a Case of Human Meningitis in Southern Italy

**DOI:** 10.1155/2024/4529326

**Published:** 2024-10-25

**Authors:** Giovanna Fusco, Saveria Dodaro, Maria Vittoria Mauro, Francesca Greco, Lorella Barca, Rubina Paradiso, Antonio Limone, Maria Garzi Cosentino, Agata Campione, Giovanna De Luca, Bianca Cecere, Sonia Greco, Valeria Vangeli, Esterina De Carlo, Giorgia Borriello, Antonio Mastroianni

**Affiliations:** ^1^Experimental Zooprophylactic Institute of Southern Italy, Portici 8055, Italy; ^2^Microbiology and Virology Unit, “Annunziata” Hub Hospital, Cosenza 87100, Italy; ^3^Infectious and Tropical Diseases Unit, “Annunziata” Hub Hospital, Cosenza 87100, Italy

**Keywords:** Italy, meningitis, phylogenetic analysis, *Streptococcus suis*, whole-genome sequencing

## Abstract

This study is the first report in Italy on the molecular characterization by whole-genome sequencing (WGS) analysis of a *Streptococcus suis* strain isolated from a human case of meningitis in Italy. The characterized *S. suis* strain was classified as a serotype 2 (SS2), multilocus sequence typing (MLST) sequence type ST1. The strain exhibited the presence of several virulence genes and resistance to penicillin, tetracycline and macrolide–lincosamide–streptogramin. Finally, we found a frameshift mutation in the gene *mrp* determining the translation of two truncated forms of the corresponding muramidase-release protein. These results highlight the importance of complete genomic data to understand the pathogenesis and epidemiology of this bacterium, capable to pose serious risks to human health.

## 1. Introduction

Meningitis is an inflammation of the meninges, the protective membranes covering the brain and spinal cord. It can be caused by different pathogens, including bacteria, viruses and fungi, the most common including *Neisseria meningitidis*, enteroviruses and *Cryptococcus neoformans* [1–3]. Bacterial meningitis is also frequently caused by streptococci like *S. pneumoniae* and *S. agalactiae*. Over the last two decades, there have been numerous reports worldwide of human cases of *Streptococcus suis*–associated diseases, leading the scientific community to recognize this bacterium as a serious threat to global public health [4]. *S. suis* is a Gram-positive, coccus-shaped zoonotic bacterium, exhibiting 29 different serotypes, including serotype 2 (SS2), which is commonly isolated in human cases of the disease [5, 6], and it is considered to be the most virulent in human cases [7], even though high virulence traits have been reported in different serotypes, although less frequently isolated [8]. In pigs, the bacterium is found as a commensal of the upper respiratory tract and of the digestive and reproductive systems [9] and is usually part of the normal microbiota of the host. However, in some cases, mostly associated with immunosuppressive events resulting from pre-existing health issues, such as chronic disease [10], recent infections and trauma [11] and alcoholism [12], this bacterium can become pathogenic, causing systemic infections, including meningitis. Additionally, it has been widely proposed that in the presence of coinfection with certain viral and bacterial pathogens, particulary those affecting the respiratory system, they enable *S. suis* to cross the mucosal barrier of the pharynx causing severe disease in both young and adult pigs [13]. The pathogenesis of this microorganism involves a complex interplay between bacterial virulence factors and host immune responses. The infection begins with the adherence to and colonization of host tissues, mediated by several surface-associated proteins, including fibronectin-binding protein (FBPS) and enolase, which binds plasminogen [14]. *S. suis* evades the host immune defences through its polysaccharide capsule which impairs opsonization, allowing the bacteria to survive and proliferate within the host. After colonization, the pathogen can invade the bloodstream leading to septicaemia and can disseminate to various organs, including the brain, joints and lungs. It also has the ability to cross the blood–brain barrier inducing meningitis. Several proteins such as muramidase-release protein (MRP), extracellular factor (EF) and specific adhesins may play roles in invasion. Once disseminated in host tissue, *S. suis* can induce inflammation and tissue damage by stimulating the production of proinflammatory cytokines. The excessive inflammatory response can result in septic shock and multiorgan failure [15, 16]. The disease is characterized by acute sepsis, meningitis, arthritis, endocarditis, pneumonia, rhinitis, abortion and vaginitis [17–19]. It is estimated that nearly 100% of pig farms worldwide harbour *S. suis* carrier animals [18]. Pigs are tipically infected at a young age, either horizontally via the “nose-to-nose” route or vertically through contact with vaginal secretions during parturition [9, 18]. In humans, the disease predominantly affects certain occupational groups such as pig farmers, veterinarians, slaughterhouse workers and butchers [4, 20]. Additionally, humans can develop the disease through handling contaminated pork-derived products, and the bacterium can enter the body through small cutaneous lesions, usually located on the hands or arms [17]. However, it is important to underline that cases of human *S. suis* infection have been reported even without the presence of skin lesions [21]. *S. suis* can also infect humans through the oral route after ingestion of contaminated raw or undercooked pork products, and only to a small extent transmission can occur via the airborne route [18]. The disease in humans can result in cases of streptococcal toxic shock-like syndrome (STSLS), meningitis, pneumonia, arthritis, endocarditis, ophthalmitis and sepsis. People who overcome meningitis often have major neurological consequences such as deafness and vestibular dysfunction. These consequences have a higher prevalence rate compared to bacterial meningitis caused by other pathogens [4, 20]. Human *S. suis* infection has a 12% fatality rate worldwide; in China, the mortality rate among patients has been reported to be as high as 18%. Meningitis is the most common clinical symptom of human *S. suis* infection and, to date, has been detected in 50%–60% of reported cases [22]. The first case of *S. suis* human meningitis was detected in Denmark in 1968, and since then, over 1600 new cases have been reported in 30 different countries globally [23, 24]. Most cases have been detected in Southeast Asia countries such as China, Thailand and Vietnam, where the disease is considered endemic due to significant pig production and high annual pork consumption per capita [24–26]. At present, in Italy, only three cases of *S. suis* human meningitis have been reported [27–29] where the strains were identified and characterized using classical biochemical and microbiological techniques. In Italy, high rates of resistance to macrolides and tetracyclines have been reported, mostly associated with the genes erm (B) and tet (M) and tet (O), respectively, in *S. suis* strains from both human and pig origin [26]. This study presents, for the first time in Italy, a case of human meningitis caused by a *S. suis* strain identified and characterized by whole-genome sequencing (WGS) analysis for virulence and antimicrobial resistance (AMR) genes.

## 2. Materials and Methods

### 2.1. Sample Collection

#### 2.1.1. Human Cerebrospinal Fluid

In January 2023, a patient approximately 60 years old was admitted at the “Presidio Ospedaliero Annunziata” hospital of Cosenza in the Calabria region of southern Italy, due to symptoms referable to meningitis. The cerebrospinal fluid was analysed by the SYSMEX XN-550 instrument following themanufacturer's instructions and displayed the following biochemical values: 7744 white blood cells/mm^3^ (85% neutrophils and 15% lymphocytes), immunoglobulin G (IgG) = 46.2 mg/dL (normal range, 0.00–3.40 mg/dL), albumin = 280 mg/dL (normal range, 0.00–35 mg/dL), andglucose = 2 mg/dL (normal range, 40–70 mg/dL). The cerebrospinal fluid was analysed for the meningoencephalitis panel by the FILMARRAYTM Meningitis/Enc kit (Biomerieux) following manufacturer's instructions and gave negative results.

#### 2.1.2. Porcine Meat Products

Following the isolation of *S. suis* from the patient's cerebrospinal fluid, veterinary staff from the competent local health authority on the 24 January 2023 conducted an inspection at the patient's home and discovered a family pig farm. During the inspection, no live animals were found, as they had already been slaughtered. Hence, only samples of pieces of muscle, liver, and sausages (during the curing phase) were collected and sent refrigerated to the “Istituto Zooproficattico Sperimentale del Mezzogiorno” (IZSM) for bacteriological examination.

### 2.2. Bacteriological Examination

Cerebrospinal fluid (CFS) sample was cultured on BD Columbia Agar with 5% Sheep Blood (Columbia, Heidelberg, Germany) and incubated in 5% CO_2_ at 37°C for 18–24 h. Typical colonies were Gram-stained, and subcultures were made from isolated colonies and used for biochemical identification using automatic VITEK 2 system (bioMérieux, https://www.biomerieux.com).

Antimicrobial susceptibility testing was carried out by the Kirby-Bauer disk diffusion method according to standard procedures. The following antimicrobials were tested: cefotaxime (30 µg), chloramphenicol (30 µg), clindamycin (2 µg), erythromycin (15 µg) and tetracyclines (30 µg). Zone interpretation criteria were conformed to human Clinical and Laboratory Standards Institute (CLSI) antimicrobial breakpoints [27]. The assessment of Minimum Inhibitory Concentration (MIC) was carried out employing the microdilution technique within 96-well microplates by using Sensititre (Thermo Scientific) instrument for the following antibiotics: ampicillin, penicillin, chloramphenicol, erythromycin, linezolid, penicillin, quinupristin/dalfopristin, tetracycline and vancomycin.

### 2.3. DNA Extraction and Sequencing

DNA was extracted from pure typical colonies, grown on TSA + blood agar plates, with the DNeasyPowerSoil kit (Qiagen) according to the manufacturer's instructions and then quantified by using the Qubit fluorometer (Thermo Fisher Scientific, https://www.thermofisher.com). Library was prepared using 150 ng of DNA using the Ion Xpress Fragment Library kit (Life Technologies, Carlsbad, CA, USA) and then loaded on Ion Chef System to prepare the template. Finally, sequencing was performed using the Ion Gene Studio S5 platform (Thermo Fisher Scientific) following manufacturer's instructions to generate 400 bp single-end reads.

### 2.4. Sequence Analyses and Phylogenetic Study

Raw data were checked for quality, trimmed by using Prinseq-lite v. 0.20.4 [28] and assembled using SPAdes genome assembler version 3.15.5 [29]. The assembled contigs were used for downstream analyses. First Kraken database [30] was used to assign taxonomic labels. Multilocus sequence typing (MLST) was performed to determine the sequence types. The allele sequences and profiles were obtained from the *S. suis* MLST database (https://pubmlst.org/ssuis). For phylogenetic analysis, all the ST1 isolates from human and animal sources were selected from GenBank database. We downloaded 1212 strains which were serotyped in silico indicating the presence of 1031 SS2 strains. All 1212 sequences were used to generate a minimum spanning tree, using the MSTreeV2 algorithm and GrapeTree for annotation. Hierarchical clustering of cgMLST (HierCC) was performed using Enterobase (https://enterobase.warwick.ac.uk/) with the cgMLSTv1 scheme for streptococci. Genes encoding for virulence factors were detected using the virulence factor database (VFDB, http://www.mgc.ac.cn/VFs/). Finally, Prokka tool [31] was used to rapidly annotate genes and identify coding sequences (CDS). AMR genes were identified using the online database ResFinder (http://genepi.food.dtu.dk/resfinder) and SsuisSerotyping pipeline [32] to assess the serotype. Draft Genome sequence of the analysed strain was deposited in GenBank with the accession number CP142676. Single-nucleotide polymorphisms (SNPs) were identified and analysed using the Snippy tool (https://github.com/tseemann/snippy.git), while mrp mutations were examined using the InterProScan tool [33].

Integrative and conjugative elements (ICEs) in *S. suis* genome were searched by using the ICEfinder bioinformatic tool [34].

## 3. Results

Bacteriological examination of the analysed samples indicated the presence of *S. suis* in the human cerebrospinal fluid sample and not in the pork-derived products collected from the patient's home. Indeed, positive cultures showed on blood agar small, greyish and alpha-haemolytic colonies, coccus-shaped, Gram-positive and Gram-negative to catalase test *S. suis* identification was confirmed by biochemical tests. The epidemiological investigation conducted by the veterinary staff revealed the absence of live animals at the patient's home as all pigs had been slaughtered during the Christmas holidays. It is a well-established tradition in many regions of Italy, including Calabria, to breed and slaughter a few pigs on a family farm to produce meat and sausage products for personal consumption. Pigs raised for self-consumption are usually slaughtered in late winter; hence, only processed and unprocessed products such as meat, liver, and sausages were found during the inspection at the patient's home. The pigs were regularly slaughtered after successfully passing the required ante- and postmortem inspection visits. *S. suis* was not isolated in the fresh pork-derived products, that is, meat and liver, nor in the cured pork-derived products. Additionally, the pork-derived products resulted negative also for *Salmonella* and *Listeria monocytogenes*.

**Figure 1:**
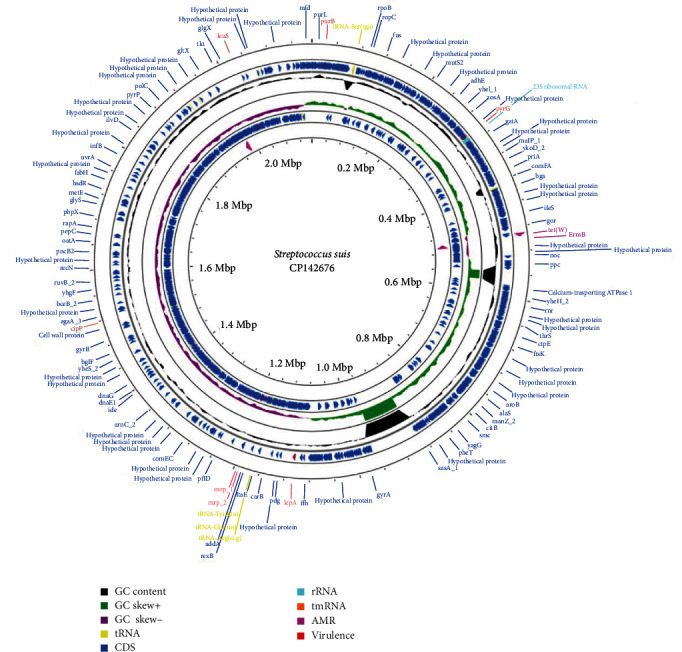
Circular representation of the whole genome of the *Streptococcus suis* strain under study. The outermost circle and the second circle show the position of the putative protein-coding genes in clockwise and counterclockwise directions, respectively. The figure shows, also, the antimicrobial genes AMR (pink), 23s rRNa (light blue), tRNA (yellow) and tmRNA (orange) and the other functional genes (blue) annotated with Prokka. The rings show finally G + C content (black) and the G/C skew information in the (+) strand (green colour) and (−) strand (dark pink colour). AMR, antimicrobial resistance; CDS, protein coding sequences; GC, guanine–cytosine; rRNA, ribosomal RNA; tmRNA, transfer–messenger RNA; tRNA, transfer RNA.

The genome of the isolated *S. suis* strain was assembled into 57 contigs longer than 200 bp. The total length of the genome was of 2,025,596 bp with a guanine–cytosine (GC) content of 41.38%. Prokka annotation showed 2013 protein CDS, 45 transfer RNA (tRNA) genes, and 5 ribosomal RNA (rRNA) genes (see Figure 1).

**Figure 2:**
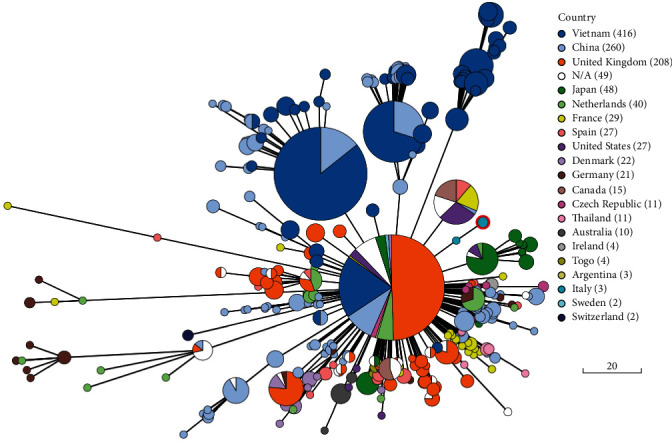
Phylogenetic analysis of all the *Streptococcus suis* whole-genome sequences available in GenBank. Minimum spanning tree of core genome multilocus sequence typing (cgMLST) was build using 1212 strains (including 1031 SS2 strains) isolated worldwide from human and animal sources. The strain under study was circled in red.

The taxonomic classification by molecular methods confirmed the identification of the analysed strain as *S. suis*. Serological classification by WGS analysis was based on the presence of specific genes. The analysis of the gene *cps2K*, coding for a specific antigenic capsular polysaccharide and associated with serotypes ½ and 2 [35], and of the virulence genes *mrp* (muramidase-release protein), *epf* (extracellular protein factor) and *sly* (suilysin), allowed to classify the strain under study as a SS2. These genes have already been used, mainly for serotype II strains, to predict the pathogenic potential of *S. suis* strains [36]. Finally, MLST characterization classified the strain as a sequence type ST1. Phylogenetic analysis performed on 1212 *S. suis* strains downloaded from the GenBank database (including 1031 SS2 strains) indicated that the analysed strains predominantly clustered by geographical origin (see Figure 2). The strains of human origin (571 total strains) were from Vietnam (317), followed by China (197), Europe (48), Togo (4), Argentina (3) and Thailand (2). The remaining strains were mostly isolated from pigs (413). At present in GenBank, there were only three whole-genome sequences of *S. suis* strains isolated in Italy: the strain under study, one isolate from a human case of blood infection in 2016 and one from a recent human neonatal case of meningitis (Fusco et al. (2024), manuscript in preparation).

**Figure 3:**
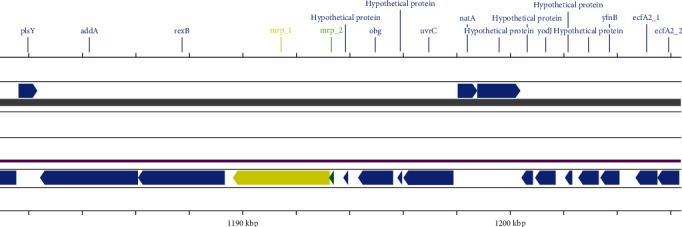
Linear representation of the chromosomal region including the sequence of the mutated *mrp* gene of the *Streptococcus suis* strain under study. The figure shows the two truncated forms of the corresponding muramidase-release protein, composed of 1210 (*mrp1*, yellow arrow) and 61 (*mrp2*, green arrow) amino acids, respectively.

To better characterize the strain under study, we investigated the presence of putative virulence factors and antibiotic resistance genes, considering only genes with ≥90% identity. The analysis revealed the presence of many additional putative virulence factors, *nleB2*, *lepA*, *pyrG*, *clpP*, *leuS and purB*, *stp1*, *ArgR*, *NisI*, *dltA*, *ABC*, *ArcC*, *PnuC*, *prsA*, *IdeS*, *ZmpA*, *ZmpB*, *ZmpC*, *SntA* and *hylA*, involved in several molecular mechanisms such as cellular metabolism, stress response and cellular regulatory systems [8]. In addition, the presence of a specific cluster of pili known to be associated with virulent strains was evaluated. The strain under study was found to have pilus-associated virulence genes including the *srtA*, *srtBCD*, *srtE* and *srtF* clusters [37]. Moreover, a frameshift mutation caused by a T insertion at nucleotide position 175 (coverage 167x) was found in the gene *mrp*. The normal structure of this protein has four domains: an N-terminal YSIRK Gram-positive signal peptide, a MucBP domain, a Surface protein repeat SSSPR-51 and a C-Terminal LPxTG cell wall anchor domain. The insertion determines the formation of a stop codon at nucleotide positions 181–183 inducing the translation of two truncated forms of the corresponding protein, composed of 61 and 1210 aminoacids, respectively (Figure 3). The two altered forms of the corresponding MRP are identified in the annotation as *mrp2*, including only the signal peptide and *mrp1*, consisting of the other three functional domains (accession number CP142676). Analysis using the InterProScan tool indicated that the identified mutation likely does not affect the functional activity of the mutated protein.

Combined results of microbiological methods indicated that the analysed *S. suis* strain was resistant to ampicillin (MIC >16 µg/ml), clindamycin, erythromycin, penicillin (MIC >16 µg/ml) and tetracycline. Moreover, it displayed intermediate susceptibility to cefotaxime. WGS data confirmed the observed resistances by identifying the presence of different genes with a significant identity, tet(W) encoding for tetracycline resistance, *erm* (B) encoding for macrolide–lincosamide–streptogramin (MLSB) resistance, ANT (6)-I responsible for resistance to aminoglycosides, folP conferring sulphonamide resistance and a number of other antibiotic resistance genes variably implicated in AMR mechanisms (Supporting Information 1: Table S1). Resistance to penicillin and nonsusceptibility to cefotaxime can be explained by the presence of a mutation in the *pbp1a* gene (encoding penicillin-binding protein 1a) consisting of the deletion of four amino acids from position 2–5 (Supporting Information 2: Figure S1) when compared with the reference sequence in the strain P1/7 (GenBank accession number AM946016) [38]. The analysis of the other known *pbp* genes (*pbp2a*, *pbp1b*, *pbp2b* and *pbp2x* genes) displayed no differences with the reference sequence. Finally, ICEs search displayed the presence of an ICE sequence (Supporting Information 3: Figure S2) belonging to the Tn916 ICE family [39] containing two AMR genes, *tet* (W) and *erm* (B).

## 4. Discussion and Conclusions

This study is the first report in Italy on the molecular characterization by WGS analysis of a *S. suis* strain isolated from a human case of meningitis. The *S. suis* strain under study was classified as a SS2, MLST sequence type ST1 and exhibited the presence of several virulence genes and resistance to penicillin, tetracycline and MLSB. Moreover, a frameshift mutation in the *mrp* gene was found, responsible for the translation of two truncated forms of the corresponding MRP.

Bacterial meningitis is a severe infectious disease that affects humans with an estimated incidence of 2.6–6 per 100,000 adults per year in developed countries [26]. Among the bacterial aetiological agents of human meningitis, *S. suis* has been more frequently documented in Asian countries and only rarely in Europe and United States [40]. The main risk factors for human *S. suis* infection are exposure to pigs or their raw meat, together with the presence of cutaneous lesions in exposed individuals. van Samkar and colleagues [26] report that 61% of human *S. suis* meningitis cases worldwide were related to direct contact to pigs. This is also the case of the present study as the patient was the owner of a family pig farm and personally handled the meat of her own animals reared and slaughtered for self-consumption. The patient did not present cutaneous lesions on hands at the time of diagnosis of *S. suis*; however, this exception has already been described previously [21], and airborne transmission of this pathogen has been demonstrated [41, 42]. The international health community displays growing interest on human *S. suis* infections due to the increasing number of human cases caused by increasing and continuous movements of humans and animals between countries worldwide. Relocated animals carry a precise microbiota and corresponding resistome selected by the peculiar pressures imposed by their originating farm management practices. Inevitably, this contributes to the spread of the AMR phenomenon, posing risks for both human and animal health. The present study is the result of an interdisciplinary collaboration between two different entities, operating for the protection of human and animal health, respectively, in accordance with the Global Action Plan on AMR drawn up by World Health Organization (WHO) and World Organisation for Animal Health (OIE) in 2016 [41]. In Italy, in 2017, the plan was adopted with the emanation of the National Plan to Combat AMR (PNCAR) 2017–2020, extended for the year 2021 and followed by the current PNCAR 2022–2025. This plan is intended to provide operational guidelines aimed at harmonizing actions of the National Health System with the international standards in a One Health perspective. Moreover, as regards *S. suis* diagnosis, collaboration between human and veterinary laboratories is desirable since many human diagnostic laboratories can be less aware of this pathogen, thus risking to misidentify it as enterococci or other species of the genus *Streptococcus* [18].

The epidemiological investigation carried out by at the patient's home revealed the presence of a small pig farm intended for self-consumption. However, by the time of the investigation, the animals had already been slaughtered for meat and sausage production. *S. suis* was not isolated in the fresh pork-derived products, that is, meat and liver, nor in the cured pork-derived products. The absence of the pathogen in the sampled meat products can be explained by the fact that the living animals were healthy at the time of slaughter and were probably only carriers of the pathogen. In carrier animals *S. suis* is part of the microbiota of the upper respiratory tract and vaginal mucosa [21]; therefore, it can be argued that the patient got infected with *S. suis* after the slaughter of the pigs and during manipulation of contaminated tissues and organs.

The *S. suis* strain under study was classified as a SS2 and MLST sequence type 1 (ST1). SS2 is the most common cause of infection in pigs in Europe and has been shown to be frequently responsible for human infections [40, 42]. SS2 has been classified into at least 16 MLST sequence types [18], including the ST1 type, highly virulent and frequently associated with invasive infections in humans worldwide [29]. In Italy, three cases of *S. suis* meningitis in humans have been reported, all caused by SS2, ST complex 1 strains [27]. Correct diagnosis and identification of the isolated strains are of utmost importance for the control of this pathogen. Indeed epidemiological data are still missing for several counties, and available data are sometimes biased by misdiagnosis of *S. suis* in the past [8, 9]. Molecular serotyping of *S. suis* is basically carried out using three virulence markers, *sly*, *mrp* and *epf*, even though the association between serotypes, sequence types and virulence of the strains is still unclear. However, these three genes still remain extensively used to predict the pathogenicity of serotype II *S. suis* strains in many countries [36]. The *S. suis* under study exhibited a mutation in the *mrp* gene but could still be classified as SS2, consistently with many reported SS2 pathogenic field strains isolated from both diseased pigs and humans exhibiting different combinations of the presence of these three genes.

The mutation identified in the *mrp* gene of the strain under study results in the production of two truncated forms of the protein. The smallest one (61 aa), including only the N-terminal portion of the protein, represented by the YSIRK signal peptide, was likely devoid of functional activity. The longest one (1210 aa) instead was composed of three functional domains of the mrp protein, including the C-terminal portion and the LPxTG cell wall anchor domain. The LPxTG motif is present in prominent virulence factors of Gram-positive bacteria, such as mrp and epf proteins, and plays a crucial role in infection by allowing the anchoring of these factors to the bacterial cell surface. Bonding of these virulence factors to bacterial cell wall requires translocation across cytoplasmic membrane, allowed by the YSIRK motif and mediated by the sortase A (srtA) enzyme. However, such sequence has not been found in all the srtA substrates and has also been found in non–cell wall proteins. Moreover, previous studies [40, 41] demonstrated that the inactivation of the YSIRK motif in the staphylococcal protein A, although diminishing protein secretion, did not affect either the anchoring ability or the functional assembly of the related protein. Furthermore, bioinformatic analysis confirmed that the identified mutation is unlikely to affect the functional activity of the mutated protein.

These evidences are consistent with the ability of the strain under study to cause meningitis in a healthy subject, not affected by immunosuppressed state or concomitant infections. Also, in Spain, a human pathogenic SS2 ST3 isolate exhibited a large variant of the *mrp* gene, identified as *mrp⁣*^*∗*^, leading to the production of a higher molecular weight protein [43]. Bae and Schneewind [40] have studied the signal peptides YSIRK-G/S, present on the surface proteins from Gram-positive bacteria, demonstrating that mutational changes did not affect their capability to interact with host cells. This evidence highlights that the virulence of this pathogen is the result of complex interactions among different virulence markers. Finally, the identification of additional putative virulence factors (*nleB2*, *lepA*, *pyrG*, *clpP*, *leuS*, *purB*, *stp1*, *ArgR*, *NisI*, *dltA*, *ABC*, *ArcC*, *PnuC*, *prsA*, *IdeS*, *ZmpA*, *ZmpB*, *ZmpC*, *SntA* and *hylA*), besides those already known to be involved in mechanisms of pathogenesis and infection, points out the importance of studying this bacterial species [44]. In this context, the WGS technique is a powerful and useful tool allowing the availability of combining data from MLST characterization and the presence or absence of virulence-associated markers at the gene level. Such information is necessary to gain insight into the pathogenesis and epidemiology of *S. suis* and improve surveillance measures for such an underestimated and under evaluated pathogen, which has been reported to be able to cause not only sporadic infections but also epidemics in both pigs and humans, with high mortality rates [45–49].

According to available data on Italian *S. suis* human isolates [27–29], the strain presented in this report displayed resistance to tetracyclines and macrolides by both phenotypic and genotypic analysis. The strain also exhibited resistance to penicillin, which is associated with a deletion of four aminoacids in the N-terminal portion of the pbp1a protein. Although the *pbp* cluster contains redundant genes, it is likely that point mutations in one or more of these genes can confer resistance to beta-lactams presumably by diminishing binding affinity with the antibiotic, as already reported for *S. pneumoniae* [9, 50]. Importantly, the strain under study exhibits a MLSb phenotype, due to the presence of the *erm* (B) gene, which has rarely been reported in human clinical isolates of streptococci [6]. *S. suis* is recognized as a reservoir of antibiotic resistance genes, able to transfer AMR genes not only to other *S. suis* clones, but also to different species of *Streptococcus* and even to other bacterial genera through horizontal gene transfer [51, 52]. Consistently with presented data, in Europe, the most frequent antibiotic resistances in *S. suis* have been reported for lincosamides, macrolides and tetracyclines. Cross-resistance among lincosamides, streptogramin B, and macrolides has often been observed since these molecules are largely used in food production animals, particularly intensive pig farming [9] and resistance determinants to these antibiotics are often linked on mobile genetic elements (MGEs) [53]. Analogously, tetracycline resistance in *S. suis* is favoured by the extensive use of this antibiotic in pig industry, due to its broad spectrum. Particularly interesting in the analysed strain is the observed resistance to penicillin, which in Italy has been described to be strictly associated with serotype 9 [54]. In Italy, the most frequent *S. suis* serotypes are 2 and 9, with serotype 9 which is progressively becoming dominant in Italy as in other European countries in the recent years [15, 54]. The transfer of AMR genes between bacteria can occur via MGEs such as transposons, insertion sequences, plasmids, prophages and chromosomal integrative elements, including ICEs and integrative and mobilizable elements (IMEs) [50]. Among these MGEs, *S. suis* predominantly uses ICEs, which contain all the necessary information for autonomous excision, conjugation and integration into the bacterial genome [39]. In the genome of the strain under study, a putative ICE belonging to Tn916 family [55] was found, carrying *erm* and *tet* genes. These data highlight the potential of our *S. suis* strain to act as an AMR gene spreader able to contribute to the acquisition, maintenance and spreading of AMR in the environment.

In conclusion, this study confirms the presence of *S. suis* SS2 ST1 as a cause of human meningitis and highlights the lack of genomic sequencing data of this pathogen in Italy. The availability of complete genomic data is essential not only for a better understanding of pathogenesis and epidemiology of this bacterium but also to improve the general capacity to diagnose, treat and prevent human infectious diseases. Indeed, genomic epidemiology approaches facilitate effective interventions by identifying transmission routes and sources, as well as monitoring genetic changes in pathogens over time. Consequently, bacterial sequencing is essential for epidemiological surveillance, controlling infectious disease outbreaks and advancing vaccine development and refinement.

## Data Availability

The genome under study can be found with accession number CP142676.

## Supporting Information

Supporting Information 1 Table S1: Antibiotic resistance genes and virulence markers identified in the *S. suis* strain under study.

Supporting Information 2 Figure S1: Mutation in the *pbp1a* gene encoding penicillin-binding protein 1a.

Supporting Information 3 Figure S2: The figure shows a putative mobile element identified (A). The putative ICE with T4SS (region 2) was in close proximity to the AMR genes tet (W) and erm (B), suggesting that these elements are part of a mobile genetic element (B).
