# Supplementary material for: First WGS Characterization of Streptococcus suis Isolated From a Case of Human Meningitis in Southern Italy
Source: Transbound Emerg Dis. 2024 Oct 25;2024:4529326. doi: 10.1155/2024/4529326 (PMC12017127; doi:10.1155/2024/4529326)
Supplement: Supporting Information 2 — Figure S1: Mutation in the pbp1a gene encoding penicillin-binding protein 1a. [file 4529326.f2.pdf]

```
penicillin-binding protein PBP1A
1   MVNLLKTTTIKKALLITANILLAGAIIACLAGAALVVYYIQSAPELTEESLTATVSSKIY 60   P1/7 seq
1   M----KTTTIKKALLITANILLAGAIIACLAGAALVVYYIQSAPELTEESLTATVSSKIY 60   CP142676
    DKNGNLIADLGAEKRSSAKTEEIPTDLVNAIVAIEDQRFFNHRGVDVIRIAGSLINNLSG
    GRLQGGSTLDQQFIKLTYFSTSVEDQNLKRKIQEAWLATQLERRNTKQEILTYYINKVYM
    SNGNYGMKTAALAFYGKELKDLTLPQLALLAGMPQAPNQYDPYTNPEDAKARRDLVLAEM
    LEEKYIDNTQYEQAVLTPVTDGLQPLSNAAAYPAYMDNYLKQVVEEVEAKTGYNLLTTGM
    DVYTNVDPAAQQQLWNIYNTDMYVSYPDDLLQVASTVVDVSNGKVVAQLGGRKQETNVSF
    GTNQAVETNRDFGSTMKPITDYAPAFENGIYTSTADLILDGPYNYPGTPTPVNNWDKQYY
    GNISVKTAIQYSRNVTAVKALEATGLENALKFLNSVGINYPDIHYSNAISSNTSDTSSKY
    GASSEKMAAAYAAFANGGTYYAPQYVNKIVFSDGTVTEYAPKGTKVMSAETAYMMTDMMK
    AVMSYGYGLNASVSGVPMAGKTGTSNYTDSETDTILASIPEANYSYMVVPDENFVGYSSQ
    YAMAVWTGYTNRMTPILDNSMRIATDVYHNMMLFMHSDYTATDWEMPSGLVRYGSNYYLR
    GSRSLSNAYNTYTSSSTSSSSSTETSETTEATTTSSETSSGTATETATSSTETATTGNNP
    ATGQTDGQ
```

Supplementary Figure 1 - Mutation in the *pbp1a* gene encoding penicillin-binding protein 1a. The mutation involves a deletion of four amino acids at the N-terminal region, specifically from positions 2 to 5. This alteration may influence the protein's interaction with penicillin.
