# Supplementary figures and images for: First WGS Characterization of Streptococcus suis Isolated From a Case of Human Meningitis in Southern Italy
Source: Transbound Emerg Dis. 2024 Oct 25;2024:4529326. doi: 10.1155/2024/4529326 (PMC12017127; doi:10.1155/2024/4529326)

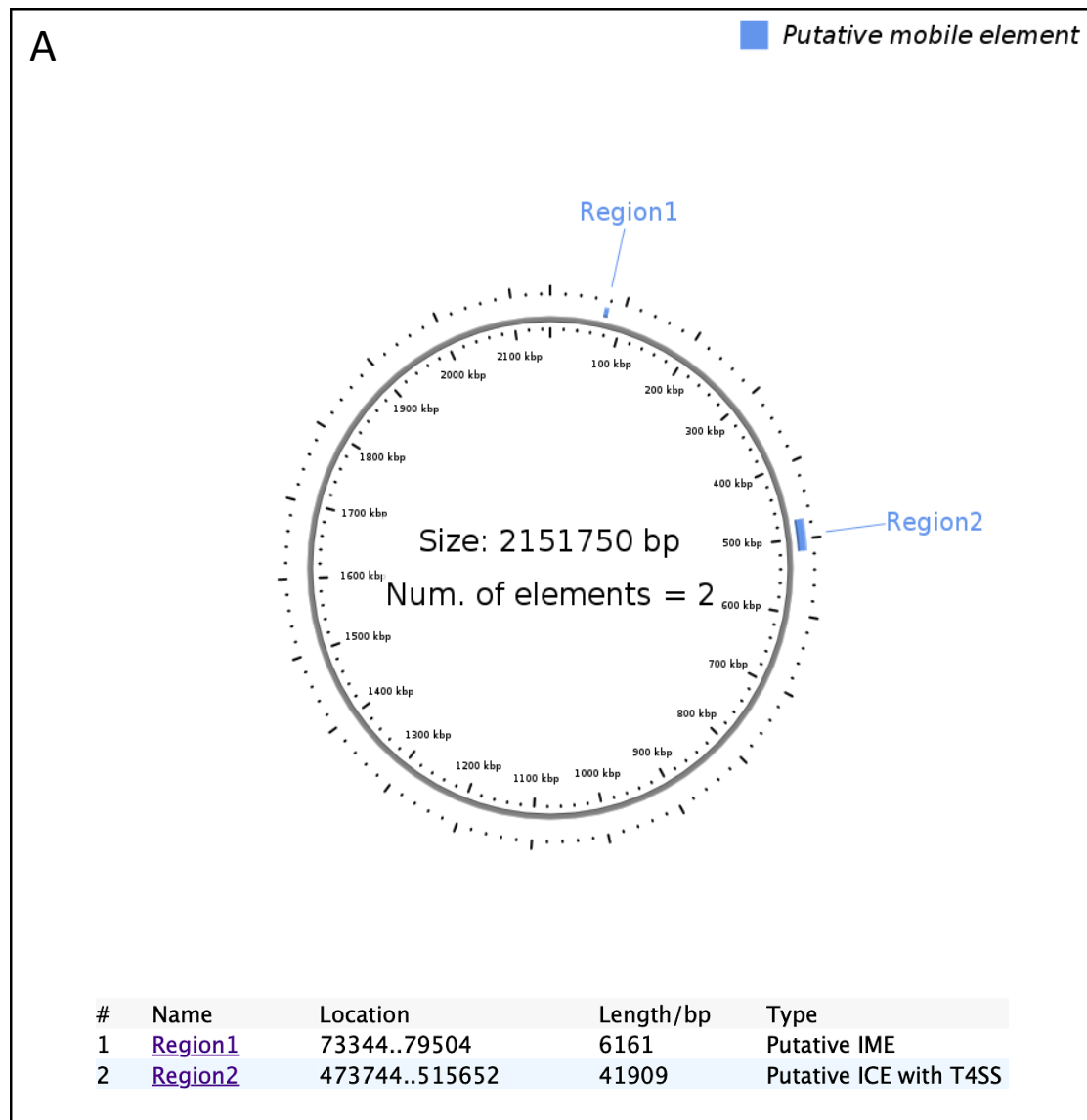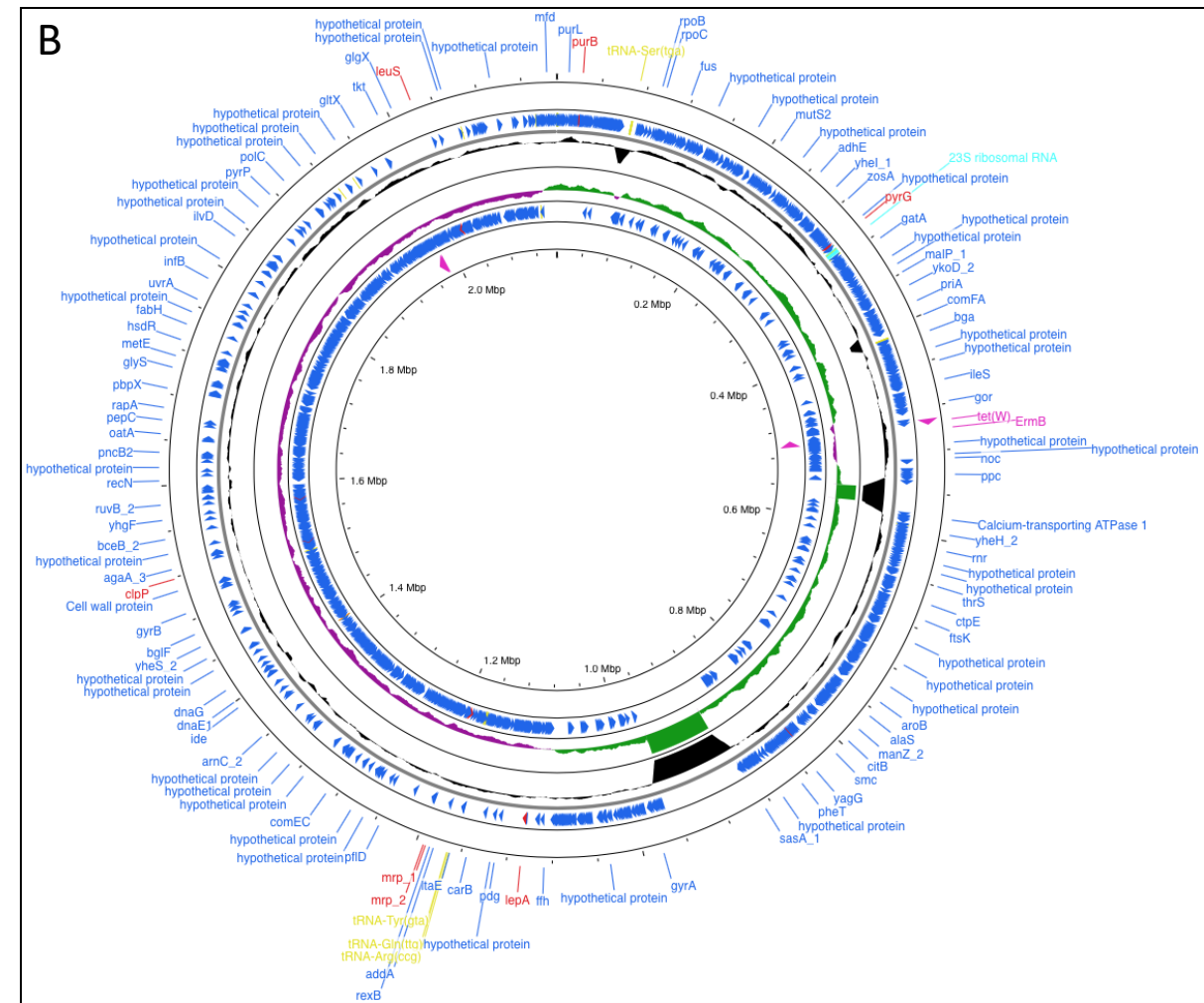

Supplement: Supporting Information 3 — Figure S2: The figure shows a putative mobile element identified (A). The putative ICE with T4SS (region 2) was in close proximity to the AMR genes tet (W) and erm (B), suggesting that these elements are part of a mobile genetic element (B). [file 4529326.f3.pdf]
